# Effect of Exposure to Air Pollution on Placental Weight in Isfahan-Iran 

**Published:** 2017-06

**Authors:** Hatav Ghasemi-Tehrani, Setare Fallah, Nafiseh Mozafarian, Sareh Miranzadeh, Shokooh Sadeghi, Azam Azidhak

**Affiliations:** 1 Department of Obstetrics and Gynecology, Isfahan University of Medical Sciences, Isfahan, Iran; 2 Isfahan University of Medical Sciences, Isfahan, Iran; 3 Department of Pediatrics, Child Growth and Development Research Center, Research Institute for Primordial Prevention of Non-Communicable Diseases, Isfahan University of Medical Sciences, Isfahan, Iran; 4 Child Growth and development Research Center, Research Institute for Primordial Prevention of Non – Communicable Diseases, Isfahan, Iran; 5 Department of Anesthesia, Shahid Beheshti Hospital, Isfahan, Iran; 6 Department of Obstetrics and Gynecology, Shahid Beheshti Hospital, Isfahan, Iran

**Keywords:** Air Pollution, Pregnancy Outcomes, Birth Weight, Placental Weight

## Abstract

**Objective:** To determine the effect of Air Quality Index (AQI) in the first trimester of pregnancy on birth weight, placental weight, and the ratio of placental weight to the birth weight (pw-bw) in Isfahan.

**Materials and methods:** This cross-sectional study was done on 312 consecutive pregnant women in Beheshti Hospital in Isfahan city in 2013. Information on air pollution was received from the Environmental department of Isfahan. Average exposure to air pollution in the first trimester of pregnancy was calculated for eachpregnant woman. In order to compare quantitative and qualitative variables, analysis of variance (ANOVA), and chi-square were applied. After that, the multiple linear regression analysis was used to assess the association the Air Quality Index (AQI) on birth weight, placental weight and the ratio of pw-bw. Potential confounders including age, baby gender, smoking of husband, maternal BMI, maternal occupation, and education and mother’s residential area were considered. A statistical significant association were considered for P-value less than 0.05.

**Results:** The findings showed that there is inverse relationship between exposure to air pollution and placental weight in the first trimester of pregnancy after controlling potential confounders (β = -2.57, p-value = 0.008). The inverse relationship between air pollution and the ratio of pw-bw was found. (β = -0.001, p-value = 0.002).

**Conclusion:** The results of this study suggest that air pollution is associated with newborns’ health which in turn is a warning alarm for considering some actions in both sides of reducing the air pollution and teaching the pregnant women about the adverse effects of air pollution on the pregnancy outcomes.

## Introduction

In recent years, air pollution is considered as one of the most important human problems from environmental stand point and its increasing rate threats human health seriously in large and industrial cities, especially in developing countries (1). Children, the elder lies, and pregnant women are more vulnerable groups when come into exposure to air pollution (2). There is a growing evidence on the adverse effects of air pollution on pregnant women such as fetal growth and pre-term delivery (3).

Preterm delivery or intrauterine growth restriction (IUGR) is the most important causes of low birth weight, infant and childhood mortality (4-6). Placental weight at birth is around 450g and it has been estimated as15% by weight of the newborn baby. Moreover, placenta growths rapidly in the early of pregnancy while the highest percentage of fetal growth is in the third trimester. With increasing of gestational age, the ratio of placental weight to fetal weight is reduced (7). Ratios of placental weight to fetal weight (PWR) indicates balance between the placenta and the fetusin addition to the changes in physiology and function (8).

There are the limited studies focused on the relationship between air pollution and placental weight. For example, Rocha e Silva et. al. who investigated on the effects of air pollution on the weight of the placenta and birth weight of mice reported that1 week exposure to air pollution causes significant reduction in the weight of the fetus. Moreover, placental weight at any stage of pregnancy in mice which were exposed to air pollution was lower compared with those were subjected to clean air.Finally, they concluded the exposure to outdoor air pollutants at any stage of pregnancy reduces birth weight and placental weight (9).Yorifuji et al.in 2010reported a direct relationship between the proximity of the location to the main routes (traffic suites routes with more vehicles) with the ratio of pw-bw. This ratio was more than 0.48 percent in the people who lived within 200 meters of the high traffic routes (10).

A prospective cohort study (2012) was shown that exposure to air pollution may effect on the functional and growth of placenta and it is associated with reduced placental weight, while it did not effect on the ratio of pw-bw (11).

Recently, the result of a study that was investigated on 116 pregnant women in Tanzania to assess the association between domestic air pollution and adverse effect on pregnancy outcomes and its potential mechanism, showed that there is relationship between exposure to air pollution (PM2.5 and CO) and fetal thrombosis that is common in low birth weight infants. However, no significant association was seen between placental weight and air pollution (12).

Until now, several studies have been conducted on the effect of air pollution on birth weight but the results are inconsistent (13, 14). Furthermore, studies have been limited about the effect of air pollution on placental weight. For these reasons as well as complications of low birth weight and increasing trend of air pollution in Isfahan, the aim of this study was to determine the relationship between air pollution with birth weight and weight of the placenta.

## Materials and methods

This cross-sectional study was approved by the Isfahan University of Medical Sciences (No: 393519) and written informed consent was obtained from all participants before sampling. Three hundred and twelve pregnant women were selected with simple sampling. Inclusion criteria were: non-smoking mother, lack of maternal chronic disease (i.e. chronic diabetes, gestational diabetes, chronic hypertension, preeclampsia, eclampsia, anemia and premature birth), multiple pregnancy, and woman who has not had more than two pregnancies. All the pregnant women have nottravelled to other places.

All samples were collected from Beheshti hospital and women who had inclusion criteria during the pregnancy as well as they settled in the range of air quality measuring station involved in this study. The required data(date of baby birth, date of first day of last menstruation (LMP), gender, age, place of residence, mother's education and her job, maternal weight before pregnancy, height, abortion or stillbirth, gravidity, a history of any of the diseases (hypertension, diabetes, hypothyroidism and hyperthyroidism, anemia and other diseases), and drug addiction)was collected by a researcher in face-to face interview.BMI was calculated from weight (kg) divided by height squared (m^2^).

At birth time, placental and birth weights were measured and recorded. After delivery, mothers were transferred to the ward and the checklist was filled out by them.

Information of six air pollution monitoring stations, including Ahmedabad, Azadi Square, Chaharbagh Khajoo, Kharazy Highway, Khomeini Shahr, and Najafabad stations in 2012-2013 were received from Environmental Department of Isfahan city; average daily air pollution was recorded according to Air Quality Index (AQI); and for each sample, it was determined for nearest location and the station data was used for the related sample. With regard to the mother living area, average exposure to air pollution in the first trimester of pregnancy (from the first day of the last menstrual period to the end of the first trimester of pregnancy) was recorded for each sample.

The statistical analysis was performed by SPSS 16 statistical program. Average exposure to air pollution in the first trimester of pregnancy were calculated for each maternal, then divided by quartiles. Upper quartile was considered as an upper level. The quantitative variables were reported as average and standard deviation and qualitative variables were as frequencies and percentages. ANOVA and chi-square tests were used for the quantitative and qualitative variables, respectively. The multiple linear regression analysis was used for relation between air pollution and birth weight, placental weight, and the ratio of pw-bw. Average exposure to air pollution in the first trimester was as independent variable and birth weight, placental weight, and the ratio of pw-bw were dependent variables which were included in the model. Potential confounders such as maternal age, maternal education, maternal BMI, drug addiction, smoking, gender, and location were considered. In all analyses, borderline statistical significance was considered to 0.05.

## Results

From 610 collected samples 312 samples were elected based on the criteria.Characteristics of study population are presented in Table 1.

The average birth and placental weights in six studied areas in Isfahan province is provided in table 2. The highest weighted average for placenta belongs for Najafabad and the lowest of that is related to the Kharrazi Highway (p-value = 0.034). Furthermore, the highest average ratio of pw-bw is related to Khomeini Shahr and the least of it is in the Kharrazi Highway (p-value = 0.006). The average exposure to air pollution during the first trimester of pregnancy based on Air Quality Index (AQI) in the investigated are as reveals that the maximum and minimum amount of exposure of pregnant women were related to Ahmedabad and Khomeini Shahr, respectively (Table 2).

**Table 1:** Characteristic of the study population

**Pregnancy outcomes**		
Low birth weight (< 2,500 g) n (%)		10 (3.2)
Birth weight Mean ± SD		3131.9 ± 331.4
Placenta weight (gr) Mean ± SD		783.97 ± 162.9
placenta / birth weight ratio Mean ± SD		0.25 ± 0.06
Covariates		
Sex of child n (%)	Boy	152 (48.7)
	Girl	160 (51.3)
Maternal smoking during pregnancy n (%)		
Addiction husband n (%)	No	283 (90.7)
	Yes	29 (9.3)
Maternal education n (%)	Primary	19 (6.1)
	Guidance	58 (18.6)
	High school	198 (63.7)
	University	36 (11.6)
Mother’s occupation n (%)	Yes	21 (6.8)
	No	286 (93.2)
Maternal age, Years Mean ± SD		27.57 ± 5.6
Maternal weight, kg Mean ± SD		65.9 ± 8.5
Maternal height, Cm Mean ± SD		161.2 ± 4.3
pre-pregnancy BMI (kg/m2)		25.3 (2.95)

As can be seen from table 3, the first quartile is less than 71.43, second quartile between 71.43 – 72.89, third quartile72.90– 82.97, and the last quartile more than 82.97 were obtained. At least facing 58.41 and a maximum of 116.75 and on average (SD) Air Quality Index (AQI) was equal to 77.48 (10.8) (Table 3).

In ANOVA test, birth weight did not show a significant difference in terms of air pollution. While, average placental weight and the average ratio of pw-bw showed significant differences in terms of air pollution. The average of both variables in the first quarter showed the highest value and lowest value in the last quarter (Table 3).

**Table 2:** Descriptive statistics for study participants in six different climatic areas in Isfahan: Mean ± SD

**Six climatic areas in Isfahan**	**birth weight(gr)**	**Placenta weight (gr)**	**placenta / birth weight ratio**	**AQI** ^*^
Ahmad-Abad Sq.(n = 36)	3175 (281.3)	771.7 (166.7)	0.245 (0.056)	99.2 (6.55)
Azadi Sq. (n = 54)	3159.3 (389.9)	805.2 (179.2)	0.257 (0.057)	74.0 (5.57)
ChaharbaghKhajoo St. (n = 39)	3137.9 (260.8)	768.3 (146)	0.25 (0.05)	77.6 (9.0)
Kharrazi Highway (n = 102)	3136.9 (243.5)	749.7 (140.3)	0.239 (0.045)	75.9 (6.6)
KhomeyniShahr (n = 47)	3036.8 (394.7)	811.7 (169.9)	0.273 (0.072)	67.6 (7.4)
Najaf Abad (n = 34)	3152 (461.7)	844.2 (184.9)	0.272 (0.063)	78.3 (4.7)
Total( n = 312)	3131.9 (331.4)	784 (162.9)	0.25 (0.057)	77.5 (10.8)

P-values are resulted from ANOVA

Multiple linear regression analysis of air pollution on birth weight, placental weight, and pw-bw ratio is presented in table 4. Results showed no significant association between birth weight and air pollution.

Multiple linear regression analysis after controlling the variables such as maternal age, maternal education, maternal BMI, baby gender, and husband smoking showed that there is an inverse correlation between air pollution and placental weight (p value =0.002) (Beta = -2.62) (model 1).

In model 2, in addition to the variables in the model 1, living place of pregnant women was controlled and a significant inverse association between air pollution and placental weight was found (p-value = 0.008) (Beta = -2.57).

By controlling all variables, theweak and significant inverse relationship between air pollution and the ratio of pw-bw was found (p-value = 0.002) (Beta = -0.001).

## Discussion

After controlling confounder variables, the results showed that there is an inverse correlation between air pollution and placental weight and increasing air pollution reduced placental weight. Also, there is a significant inverse relationship between air pollution and the ratio of pw-bw. Furthermore, nosignificant relationship between air pollution and birth weight was found.

Although in recent decade several studies reported on the relationship between air pollution and low birth weight in the world.(13, 15-17), but the relationship between two mentioned factors seems controversial. 

**Table 3:** Characteristic of mothers and neonatal by Air Quality Index (AQI) quartile for first trimester of Pregnancy

**Characteristic**		**Total**	**Quartile 1** **< 71.435**	**Quartile 2** **71.435-72.89**	**Quartile 3** **72.9-82.97**	**Quartile 4** **> 82.97**	**P value**
Number (%)		312(100)	78 (25)	82 (26.3)	79(25.3)	73(23.4)	
Placenta weight (gr) ¶		783.97 (162.9)	836.54 (169.96)	773.09 (137.85)	790.10(151.44)	732.78 (178.22)	0.001*
birth weight(gr) ¶		3131.9 (331.4)	3080.4(408.04)	3147.2 (261.6)	3144.9(341.6)	3155.6 (298.2)	0.47*
placenta / birth weight ratio¶		0.25(0.06)	0.28(0.07)	0.246 (0.045)	0.253(0.05)	0.23 (0.06)	< 0.001*
Maternal Age(years) ¶		27.57 (5.6)	28.78 (5.9)	26.59 (5.02)	26.95(5.12)	28.04 (6.3)	0.055*
Pre-pregnancy BMI(kg/m2)¶		25.27 (2.95)	25.31 (3.16)	25.6 (2.78)	25.53(3.1)	24.55 (2.65)	0.11*
Sex of child©	Boy	152 (48.7)	35(44.9)	45(54.9)	35(44.3)	37 (50.7)	0.48‡
	Girl	160 (51.3)	43(55.1)	37(45.1)	44(55.7)	36 (49.3)	
Mother’s occupation ©	Yes	21(6.8)	6(7.7)	7(8.8)	4(5.2)	4 (5.6)	0.79‡
	No	286(93.2)	72(92.3)	73(91.2)	73(94.8)	68 (94.4)	
Mother’s education ©	Primary	19 (6.1)	6 (7.7)	5 (6.1)	4 (5.1)	4 (5.5)	0.058‡
	Guidance	58 (18.6)	13 (16.7)	19 (23.2)	13 (16.7)	13 (17.8)	
	High school	198 (63.7)	41 (52.6)	51 (62.2)	56 (71.8)	50 (68.5)	
	University	36 (11.6)	18 (23.1)	7 (8.5)	5 (6.4)	6 (8.2)	

* P-values are resulted from ANOVA,

‡ P-values are resulted from Chi-square test;

¶ Data are expressed as Mean (SD);

© Number (percentages)

**Table 4:** Multiple linear regression analysis of air pollution on birth weight, placental weight and pw-bw ratio

			**Birth weight**	**Placenta weight**	**Placenta / birth ** **weight ratio**
Air Quality Index (AQI)	Crude models	Beta	1.93	-2.73	-0.001
		p-value	0.26	<0.001	<0.001
	Model 1^a^	Beta	2.36	-2.62	-0.001
		p-value	0.162	0.002	<0.001
	Model 2^b^	Beta	1.89	-2.57	-0.001
		p-value	0.324	0.008	0.002

a Model 1 adjusted for: Maternal Age ، mother’s education, Mother’s occupation, Maternal BMI,Addiction husband, Sex of child

b Model 2 adjusted for: Maternal Age ، mother’s education, Mother’s occupation, Maternal BMIAddiction husband, Sex of child, area

For example, a study reported in Norway on 17533 mothers and sons showed an association between exposure to air pollution during pregnancy with birth weight, these results are in agreement with our results, however the significant correlation was not found at their study’s after adjusted with confounding variables such as maternal weight (18). Moreover, in another study in Norway (when adjusted for confounding variables) no significant relationship was reported between air pollution and birth weight which is not in agreement with our results (14). A nationwide cohort study on75166 newborn babies in Denmark in 2016 showed that there was no significant relationship between birth weight and air pollution as our results were showed (19). 

Although in several studies a significant positive correlation between air pollution and birth weight has been reported. For example, a study in Beijing showed that the sulfur dioxide and particulate matter in the air increase the risk of underweight among the infants (13). Moreover, a cross-sectional study (2000) investigated on 108173 newborn babies in Czechoslovakia showed a relationship between the exposure with air pollution (sulfur dioxide and particulate matter partly) in the pregnant women with the low birth weight and prematurity (16). An other study was conducted on pregnant women who lived on the suburbs of petrochemical factories showed the carbon monoxide during pregnancy reduces fetal weight especially in the second trimester (20). A similar study performed in six polluted provinces of America showed that babies who were affected by carbon monoxide environment in the last trimester of embryonic had lower weight than those who were not exposed (21). That is inconsistent with present study. However, in this study only investigated the exposure to air pollution in the first trimester. Also, the type of pollution is not considered in this study.

In addition, many variables such as genetics, maternal weight, maternal lifestyle variables, socioeconomic status, medical conditions before or during pregnancy, smoking during pregnancy can effect on birth weight (22). Despite considering of the most confounding variables in this study, but some other important variables, such as lifestyle, particularly nutritional status of mother, number of parental care visits, and socioeconomic status could effect on the results, still should take into account for more studies. Therefore it is suggested to do future studies that should evaluate the remaining variables and controlled as a confounding.

In this study, a significant inverse relationship between air pollution, placental weight, and the ratio of pw-bw was found. The reported results in this research area found to be controversial. For example aprospective cohort study (2012) on 7801 pregnant women in the Netherlands showed that exposure to air pollution may also have an impact on the growth of the placenta and its results confirmed that exposure to air pollution is associated with reduced placental weight, while, the ratio of pw-bw are not influenced (11). One study one in the city of Poitiers and Nancy (France) (2012) showed the relationship between air pollution and placental weight and placental to fetal weights(PFR) in Nancy city but this relationship was not seen in the city of Poitiers (23). Also, a recent study in Tanzania found a significant association between placental weight and air pollution (12). While another cohort study (2016) in Denmark reported a significant positive correlation between air pollution and placental weight (19). It should be noted that these studies may be different in measurement of air pollution, the type of contamination, the exposure of pregnant women with air pollution, the type of controlled turbulence, using different statistical methods, and sample size. As a result the findings of the study hurt each other.

Potential mechanism that explains the reason of air pollution influence on the preterm birth and low birth weight is placental function failure in the transfer of oxygen and nutrients through the placenta. Its reasons are oxidative stress, inflammation, coagulation, inefficient endothelial function, and hemodynamic responses. Also, the epidemiologic studies confirm this theory that air pollution effects on the results of birth via inflammation and endothelial dysfunction. However, according to our knowledge, no study on the effect of air pollution on the placenta (the transfer of oxygen and nutrients by placenta) has been performed. Oxygen and nutrients are transferred between mother and fetus by placenta. The ratio of pw-bw is known as a biological marker for detection placenta transmission efficiency and is considered as a predicting index for result of delivery. Previous studies have revealed that malnutrition can induce compensatory growth of the placenta to the fetus birth weight. Similarly, smoking and anemia disorder the function of the placenta to transport nutrients and oxygen. Also, they increase the ratio of pw-bw (10).

Control of confounding variables such as age of the mother, and maternal education, maternal weight before pregnancy, maternal height and weight, smoking partner and gender, and the location are the strength points of this study. The social economic factors and lifestyle were controlled partly by controlling the location of pregnant women.

***Conclusion:*** Based on the conducted analysis, it seems that there is a significant relationship between air pollution and reduced of placental weight and pw-bw ratio. Interventional studies to reduce the pollutants and its effects on birth weight and placental weight is recommended. Also, more effective strategies for controlling air pollution to be considered. On the other hand, the pregnant women should be taught about the adverse effects of air pollution on pregnancy outcomes. Pregnant women are warned for less transit in contaminated areas and crowded into the city during pregnancy.
